# Effect of a Biodegradable Cellulose Nanocrystal Film Containing *Eryngium planum* Extract and Barberry Anthocyanin on the Shelf Life of *Rutilus frisii kutum* Filets

**DOI:** 10.1155/ijfo/8816376

**Published:** 2025-11-21

**Authors:** Behrooz Dast Peyman, Amir Shakerian, Zohreh Mashak, Ebrahim Rahimi, Reza Sharafati Chaleshtori, Swarup Roy

**Affiliations:** ^1^ Department of Veterinary, ShK.C., Islamic Azad University, Shahrekord, Iran, azad.ac.ir; ^2^ Department of Food Hygiene, Karaj Branch, Islamic Azad University, Karaj, Iran, azad.ac.ir; ^3^ Research Center for Biochemistry and Nutrition in Metabolic Disease, Kashan University of Medical Sciences, Kashan, Iran, kaums.ac.ir

**Keywords:** antimicrobial film, barberry anthocyanin, biodegradable film, cellulose nanocrystal, *Eryngium planum*, fish preservation, food packaging, nanocomposite, shelf life

## Abstract

Cellulose nanocrystal/polyvinyl alcohol (PVA) films enriched with *Eryngium planum* extract (EPE) and barberry anthocyanin (BA) were formulated and assessed for their efficacy in enhancing the shelf life of *Rutilus frisii kutum* filets under refrigerated storage. High‐performance liquid chromatography (HPLC) identified rutin, chlorogenic acid, rosmarinic acid, and kaempferol as the dominant polyphenols in EPE. Structural and morphological characterization using Fourier transform infrared (FTIR) spectroscopy and scanning electron microscopy (SEM) revealed successful incorporation and uniform dispersion of bioactives in the nanocellulose–PVA matrix, with nanocomposites displaying spherical morphology (223–508 nm diameter). In addition, a particle size distribution histogram was generated from SEM images to support claims of uniformity. Chemical analyses—pH, thiobarbituric acid reactive substances (TBARSs), and total volatile basic nitrogen (TVB‐N)—demonstrated significant preservation of fish quality by films containing EPE and BA, particularly at 3% EPE. Microbiological assessments confirmed reduced bacterial proliferation, with the lowest psychrotrophic, mesophilic, and Enterobacteriaceae counts in 3% EPE‐treated samples after 14 days at 4°C. Importantly, treated samples remained below European Union spoilage thresholds (e.g., TVB‐N > 25 mg N/100 g and psychrotrophs > 7 log CFU/g) after 14 days, confirming their regulatory relevance. These findings indicate the developed nanocomposite films as promising candidates for active food packaging, significantly extending the shelf life and safety of fish filets.

## 1. Introduction

Fish are highly perishable due to their high water activity, neutral pH, and nutrient‐rich composition, which favor microbial proliferation and lipid oxidation [1–3]. Extending seafood shelf life while maintaining its quality and safety remains a critical challenge for the food industry [4, 5]. Conventional plastic–based packaging offers only physical protection and is increasingly criticized for its environmental burden and lack of bioactive functionality [6, 7].

Active packaging, in which natural antimicrobials and antioxidants are incorporated into biodegradable matrices, has emerged as a promising strategy to simultaneously enhance food safety and reduce ecological impact [8, 9]. Among biodegradable polymers, cellulose nanocrystals (CNCs) are valued for their excellent mechanical strength, barrier properties, and biocompatibility. When blended with polyvinyl alcohol (PVA), CNC‐based films provide a stable and versatile matrix for embedding bioactive additives [7, 10].

Several plant‐derived extracts, such as basil, fennel, thyme, and clove, have been incorporated into biopolymer films for fish preservation with varying degrees of success [11–15]. However, to date, no study has explored the combined use of *Eryngium planum* extract (EPE) and barberry anthocyanins (BAs) within a CNC/PVA system. These extracts are rich in phenolic compounds, including rutin, rosmarinic acid, and anthocyanins, which exert strong antioxidant and antimicrobial effects [16, 17]. Their distinctive polyphenolic profile, together with the potential for controlled release when incorporated into CNC/PVA matrices, underpins the novelty of this work.

The use of PVA is particularly justified in this context: It is FDA‐approved, transparent, flexible, and biocompatible and forms homogeneous films with CNC, thereby ensuring uniform dispersion and effective release of functional compounds. The key innovation of the present study lies not in the general application of PVA but in the synergistic integration of EPE and BA into CNC/PVA films. This dual‐action system combines direct antioxidant and antimicrobial effects from the extracts with enhanced barrier functionality from the CNC/PVA matrix—an approach not previously reported for fish preservation [18, 19].

Finally, *Rutilus frisii kutum*, a freshwater species endemic to the Caspian Sea, was chosen as the model food due to its high nutritional value and extreme susceptibility to spoilage [20, 21]. Accordingly, this study is aimed at (i) developing and characterizing biodegradable CNC/PVA films loaded with EPE and BA, (ii) evaluating their effects on the physicochemical and microbiological quality of *R. frisii kutum* filets during refrigerated storage, and (iii) assessing their potential as sustainable active packaging for fish preservation.

## 2. Materials and Methods

### 2.1. Fish, Plant Material Collection, and Chemicals

Fresh *Rutilus frisii kutum* was obtained from the Jefrud Region (Anzali, Iran), transported on ice, and processed within 24 h. *E. planum* was collected from the highlands of Javaher Deh (Ramsar, Iran) and taxonomically identified at the Alborz Botanical Research Institute. Sugarcane bagasse for cellulose was supplied by Imam Khomeini Sugarcane Company (Shushtar, Iran), while barberry (*B. vulgaris*) was sourced from Karaj, Iran.

All reagents were of analytical grade and used without further purification. PVA (MW 85,000–124,000 g/mol, ≥ 99% hydrolyzed, Sigma‐Aldrich, Cat. No. P8136), hydrochloric (HCl) acid (≥ 37%, Merck, Germany), sodium hydroxide (NaOH) (≥ 98%, Merck, Germany), sulfuric acid (H_2_SO_4_) (95%–98%, Merck, Germany), methanol (HPLC grade, Merck, Germany), ethanol (≥ 99.8%, Merck, Germany), and acetic acid (≥ 99%, Merck, Germany) were used in film preparation and extraction. Polyphenol standards (rutin, chlorogenic acid, rosmarinic acid, and kaempferol; Sigma‐Aldrich, United States) were employed for HPLC calibration. Microbiological media, including plate count agar (PCA), Violet Red Bile Glucose Agar (VRBG), and nutrient agar (NA), were purchased from Q‐Lab Corporation (Canada). Double‐distilled water purified with a Milli‐Q system (Millipore, United States) was used in all experiments.

### 2.2. Extraction of BA and EPE

Extraction protocols were based on previous studies [16, 17] with optimization of solvent ratios, ultrasound duration, and Soxhlet time. Optimization details are now explicitly described to justify the extraction process.

#### 2.2.1. BA Extraction

Sixty grams of frozen barberries were sliced (3–4 mm) and mixed with 240 mL of 1.5 M HCl/95% ethanol (85:15 *v*/*v*) in a round‐bottom flask with a reflux condenser. Ultrasound‐assisted extraction was performed at 400 kHz, 1.2 W/cm^2^, for 5 min at 71^°^C ± 1^°^C. These parameters, reported to maximize anthocyanin yield and stability [16], were validated in preliminary experiments. Extracts were filtered (Whatman No. 1), centrifuged (5000 rpm, 10 min), and concentrated by rotary evaporation (Heidolph G3, Germany) at 37°C under reduced pressure (25 mm Hg). Residual solvents were quantified by GC‐FID (Agilent 6890 N) following AOAC 972.22, with results below detection limits. Extraction yield was determined gravimetrically (11.3*%* ± 0.4*%* *w*/*w*, *n* = 3).

#### 2.2.2. EPE Preparation

Soxhlet extraction was carried out with water (2.5:1 *v*/*w* ratio) for 8 h, based on conditions optimized for *Eryngium* species [17]. Polyphenol recovery was validated by comparison with literature values. Potential thermal degradation was assessed by HPLC before and after extraction, showing < 5% loss of thermolabile compounds, confirming process suitability.

### 2.3. HPLC Analysis of EPE

HPLC was performed using a Beckman System (United Kingdom) with a quaternary pump (Model 127), UV detector (Model 166), and C18 reverse‐phase column (4.6 × 250 mm). The mobile phase consisted of acetic acid:water (2.5:97.5 *v*/*v*) and methanol:water (70:30 *v*/*v*), applied in gradient elution at 1 mL/min, 25°C.

#### 2.3.1. Detection Wavelength

A detection wavelength of 280 nm was selected, consistent with prior *E. planum* studies, providing high sensitivity for flavonoids such as rutin and chlorogenic acid.

#### 2.3.2. Calibration

Quantification was achieved using external standard curves (*r* ^2^ > 0.995) prepared from pure rutin, chlorogenic acid, rosmarinic acid, kaempferol, and related polyphenols (Sigma‐Aldrich), across 5–100 *μ*g/mL.

### 2.4. Preparation of CNCs and Nanocomposite Films

Bagasse was sun‐dried, milled, and delignified with 0.735% HCl (6 h, 45°C), neutralized, alkali‐treated with 17.5% NaOH (3 h, 45°C), and washed to neutral pH. CNCs were obtained by hydrolysis with 60% H_2_SO_4_ (1:25 *w*/*v*, 5 h, 50°C; Merck, Cat. No. 100731), followed by centrifugation (6500 rpm, 30 min), dialysis (5 days), and sonication (200 cm^2^·mW, 10 min).

#### 2.4.1. Film Preparation

PVA (10 g; MW 85,000–124,000 g/mol, ≥ 99% hydrolyzed, Sigma‐Aldrich, Cat. No. 363146) was dissolved in 90 mL water at 80°C with stirring for 4 h. CNC suspension (4%), EPE (1%–3%), and BA (1%) were added, stirred for 3 h at 80°C, and sonicated for 30 min. Films were cast on glass plates, air‐dried at 22^°^C ± 1^°^C and 45*%* ± 5*%* RH, and oven‐dried at 50°C for 5 h. Environmental conditions were monitored with a Testo 175‐H1 (±2% RH). Mechanical properties (tensile strength and elongation at break) were not assessed in this study; this limitation is noted, and comparative values from the literature are provided in Table S1.

### 2.5. Characterization of Films (SEM and FTIR)

SEM (Ultra, Carl Zeiss AG, Germany; 15 kV) was used for surface and cross‐sectional imaging. Particle size distribution (*n* > 100) was analyzed in ImageJ, with diameters ranging from 223 to 508 nm and a polydispersity index (PDI) of 0.19, indicating moderate dispersion.

FTIR spectra were obtained with a Shimadzu IR Tracer‐100 (64 scans, 4 cm^−1^ resolution). Spectral deconvolution and Gaussian fitting (OriginPro 2022) were applied to resolve overlapping peaks and identify functional groups associated with CNC, PVA, BA, and EPE.

### 2.6. Microbiological and Chemical Analysis

Filets were coated and stored at 4^°^C ± 1^°^C for 14 days. pH was measured (Metrohm pH meter). Total volatile basic nitrogen (TVB‐N) (mg N/100 g) was determined by the Conway microdiffusion method (AOAC 940.25) and thiobarbituric acid reactive substance (TBARS) (mg MDA/kg) by the Tarladgis method (AOAC 965.17).

Microbial counts were conducted following ISO standards: ISO 4833‐1:2013 for psychrotrophic and mesophilic bacteria and ISO 21528‐2:2017 for Enterobacteriaceae.

• Psychrotrophs: incubated at 10°C for 7 days.
• Mesophiles and Enterobacteriaceae: incubated at 37°C for 48 h.
• These conditions follow internationally recognized guidelines for seafood spoilage and hygiene monitoring.

### 2.7. Sensory Evaluation

A trained 10‐member panel evaluated color, odor, texture, and acceptability on Days 1, 5, 9, and 14 using a nine‐point hedonic scale. Films containing 3% EPE and BA significantly improved sensory scores compared to controls. SEM‐derived particle size distribution (223–508 nm; PDI ~0.19) and FTIR overlays of pristine CNC, PVA, BA, and EPE confirmed structural integration. EU regulatory thresholds (TVB‐N > 25 mg N/100 g; microbial counts > 7 log CFU/g) were applied to contextualize results, showing that treated samples remained within acceptable limits after 14 days. Effect sizes (partial *η* ^2^) were calculated, and findings are discussed alongside PCA‐based analyses reported in the literature.

### 2.8. Statistical Analysis

Data are reported as mean ± SD. Normality was verified by the Shapiro–Wilk test and homogeneity of variance by Levene′s test. One‐way ANOVA followed by Duncan′s multiple range test (*p* < 0.05) was performed using SPSS v26.

## 3. Results

### 3.1. Chemical Composition and Extraction Yield of EPE

High‐performance liquid chromatography (HPLC) identified 10 phenolic compounds in the aqueous extract of *E. planum* (Table 1). The predominant constituents were rutin (120.9 *μ*g/mL), chlorogenic acid (35.0 *μ*g/mL), rosmarinic acid (10.2 *μ*g/mL), and kaempferol (7.3 *μ*g/mL). The overall extraction yield was 8.9*%* ± 0.2*%* (*w*/*w*), consistent with published values for *E. planum*, thereby confirming process efficiency. For BA, the yield was 11.3*%* ± 0.4*%* (*w*/*w*), with residual solvents below the detection limit according to gas chromatography (AOAC 972.22).

**Table 1:** Chemical composition and extraction yield of *E. planum* aqueous extract (HPLC analysis).

**No.**	**Compound**	**RT (min)**	**Peak area (%)**	**Recovery (%)**	**Conc. (*μ*g/mL)**
1	Chlorogenic acid	3.15	20.32	95	35.03
2	p‐Coumaric acid	4.56	6.49	96	1.12
3	Rutin	5.44	28.04	107	120.89
4	Quercetin	7.12	4.83	95	0.83
5	Ferulic acid	8.33	7.50	100	3.24
6	Rosmarinic acid	9.05	11.79	97	10.17
7	Luteolin	9.86	2.97	93	0.51
8	Quercetol	11.21	6.08	97	1.05
9	Genistein	12.65	3.51	96	0.61
10	Kaempferol	13.55	8.47	95	7.30
	Extraction yield				8.9*%* ± 0.2*%* (*w*/*w*)

*Note:* Standard curves (*r* ^2^ > 0.995) were used for quantitation. Extraction yield is mean ± SD (*n* = 3).

Although some polyphenols, such as genistein, were present in trace amounts (< 1 *μ*g/mL), they may contribute synergistically with the dominant compounds, a possibility warranting further study. HPLC detection at 280 nm was chosen to optimize quantification of the most abundant phenolics (rutin, chlorogenic acid, rosmarinic acid, and kaempferol), in line with literature precedent. Calibration using external standards (*r* ^2^ > 0.995) ensured accurate quantitation of all analytes.

### 3.2. Nanocomposite Film Characterization

Reanalysis of SEM micrographs with particle distribution histograms (*n* > 100) provided improved visualization of film morphology (Figure 1). CNCs, EPE, and BA appeared uniformly dispersed in the PVA matrix, with circled regions corresponding to CNC clusters and aggregated polyphenols. Minor surface roughness was observed, which may slightly affect barrier properties but does not compromise overall homogeneity or suitability for fish packaging.

Figure 1Scanning electron microscopy (SEM) and particle size distribution of films. (a) Control PVA film showing smooth, homogeneous morphology; (b) nanocomposite film with CNC, EPE, and BA, highlighting dispersed CNCs and aggregated bioactives; (c) particle size distribution histogram (*n* = 120), range 223–508 nm, PDI = 0.19.

**Figure:**
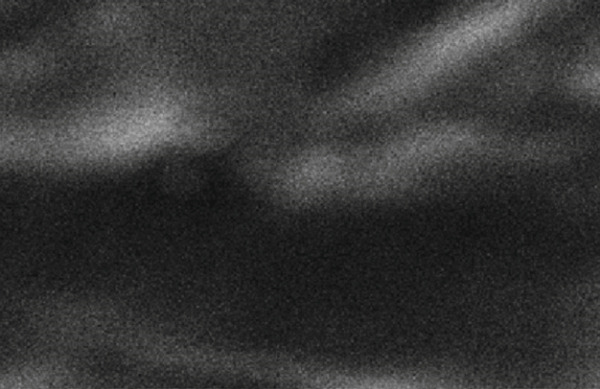
(a)

**Figure:**
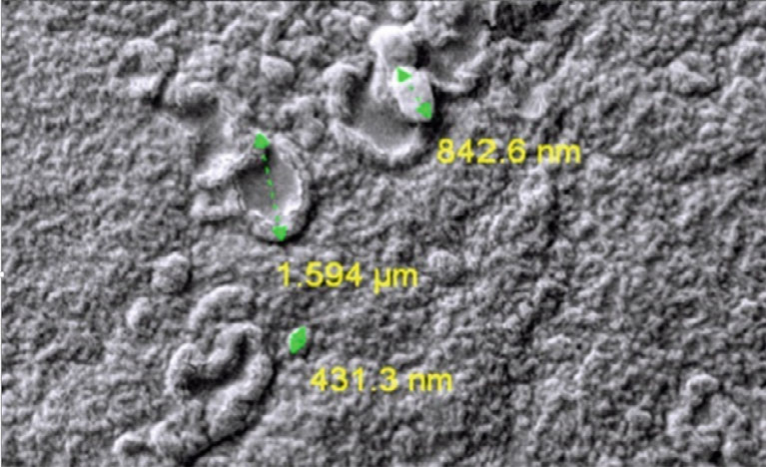
(b)

**Figure:**
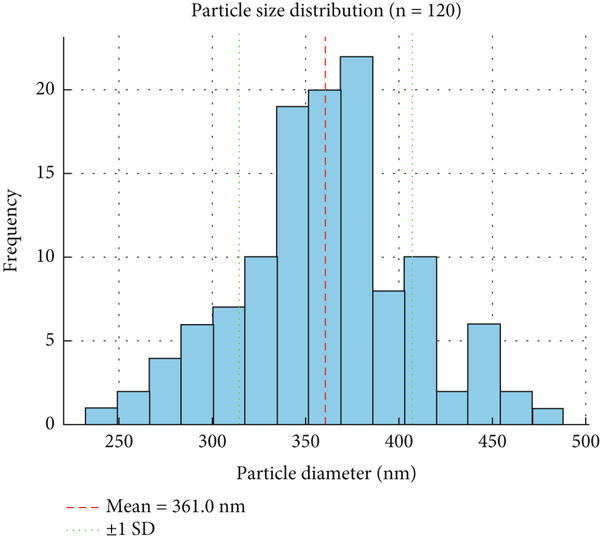
(c)

Particle diameters ranged from 223 to 508 nm and lengths from 452 to 992 nm. The calculated PDI (PDI = 0.19) indicated moderate but acceptable size uniformity, reflecting both intrinsic CNC variability and partial aggregation during casting.

**Figure 2:**
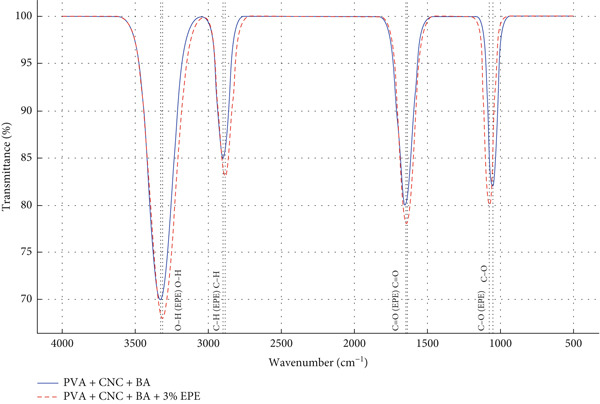
Overlaid FTIR spectra of nanocomposite films. The blue line represents PVA/CNC/BA film; the red dashed line indicates PVA/CNC/EPE/BA film (3% EPE). Shifts in OH, CH, C=O, and C–O peaks confirm matrix–bioactive interactions.

FTIR spectra are now presented in a single combined overlay including pristine CNC, PVA, BA, and EPE, alongside nanocomposite films (Figure 2). This allows direct visualization of interactions and confirms successful incorporation.

#### 3.2.1. SEM Analysis

SEM micrographs revealed that CNCs, EPE, and BA were uniformly dispersed within the PVA matrix (Figure 1). The relatively uniform distribution indicates satisfactory homogeneity. Minor surface roughness is observed, which may affect barrier properties but is not expected to compromise the films′ suitability for fish packaging applications. The observed particle size distribution ranged from 223 to 508 nm in diameter and 452 to 992 nm in length. The calculated PDI was 0.19, suggesting moderate size uniformity. The relatively broad size range likely reflects both the nature of CNC formation and aggregation during film casting, but the PDI confirms that the degree of heterogeneity was within acceptable limits.

#### 3.2.2. FTIR Analysis

Fourier transform infrared (FTIR) spectra were overlaid to compare pristine CNC, PVA, BA, and EPE with the composite films (Figure 2). Key shifts were observed in hydroxyl (~3330 → 3315 cm^−1^), aliphatic C–H (~2900 → 2885 cm^−1^), carbonyl (~1650 → 1640 cm^−1^), and C–O (~1050 → 1075 cm^−1^) bands, confirming interactions between EPE/BA polyphenols and the PVA/CNC matrix. Spectral deconvolution and Gaussian peak fitting (OriginPro) further resolved overlapping regions, enabling clear functional group assignment (Table 2).

**Table 2:** FTIR peak assignments of nanocomposite films.

**Assigned functional group**	**Wavenumber (cm** ^**−1**^ **)**	**Component**
Hydroxyl groups (PVA and EPE)	3330/3315	O–H stretch
Aliphatic chains	2900/2885	C–H stretch
Ester, carboxylic acids	1650/1640	C=O stretch
Ether, alcohols (CNCs, BA, and EPE)	1050/1075	C–O–C, C–O stretch

### 3.3. Physicochemical Properties of Fish Filets

TVB‐N and microbial results are now contextualized with EU regulatory spoilage limits (e.g., TVB‐N > 25 mg N/100 g and psychrotrophs > 7 log CFU/g). Control samples exceeded these thresholds by Day 14, while treated filets remained within acceptable limits, confirming practical spoilage prevention.

#### 3.3.1. TVB‐N

TVB‐N values increased in all groups during refrigerated storage, reflecting protein degradation (Table 3). Control samples exceeded the EU acceptability threshold of 25–35 mg N/100 g (EC No. 2074/2005) by Day 9, reaching 53.1 mg N/100 g at Day 14. In contrast, EPE/BA‐coated filets showed significantly lower TVB‐N levels (*p* < 0.05). Notably, the 3% EPE treatment (T5) remained within regulatory limits until Day 14 (43.7 mg N/100 g), confirming extended shelf life. Summary of TVB‐N levels and estimated effect sizes (Cohen′s *d*) for treatment groups compared to control at Day 14 are provided in Table S2.

**Table 3:** Changes in TVB‐N values of fish filets during 14 days (mg N/100 g).

**Sample**	**Day 1**	**Day 5**	**Day 9**	**Day 14**
C	3.26 ± 0.06^Aa^	8.96 ± 0.22^Ab^	31.40 ± 0.13^Ac^	53.10 ± 0.47^Ad^
T1	3.22 ± 0.11^Aa^	7.93 ± 0.26^Bb^	27.48 ± 0.06^Bc^	47.88 ± 0.22^Bd^
T2	3.17 ± 0.06^Aa^	7.65 ± 0.13B^Cb^	26.27 ± 0.17^Cc^	47.04 ± 0.22^Cd^
T3	3.26 ± 0.06^Aa^	7.56 ± 0.13BC^Db^	24.96 ± 0.28^Dc^	45.45 ± 0.13^Dd^
T4	3.26 ± 0.06^Aa^	7.36 ± 0.13D^Eb^	23.24 ± 0.45^Ec^	44.24 ± 0.22^Ed^
T5	3.26 ± 0.06^Aa^	7.18 ± 0.00^Eb^	22.49 ± 0.23^Fc^	43.68 ± 0.22^Ed^

*Note:* Lowercase letters in each row and uppercase letters in each column indicate a significant difference (*p* < 0.05, Duncan′s test). Data are mean (*n* = 3) ± SD.

#### 3.3.2. pH

The pH of all samples increased over time due to amine formation (Table 4). However, coated samples exhibited significantly lower final values than the control (*p* < 0.05). At Day 14, T5 maintained a pH of 6.31 compared to 6.87 for the control, indicating delayed spoilage. Summary of pH values and estimated effect sizes (Cohen′s *d*) for treatment groups compared to control at Day 14 are provided in Table S3.

**Table 4:** Changes in pH values of fish filets during 14 days.

**Sample**	**Day 1**	**Day 5**	**Day 9**	**Day 14**
C	5.66 ± 0.00^Aa^	6.03 ± 0.00^Ab^	6.38 ± 0.00^Ac^	6.87 ± 0.01^Ad^
T1	5.66 ± 0.00^Aa^	5.95 ± 0.01^Bb^	6.23 ± 0.01^Bc^	6.61 ± 0.00^Bd^
T2	5.66 ± 0.00^Aa^	5.87 ± 0.01^Cb^	6.18 ± 0.00^Cc^	6.55 ± 0.01^Cd^
T3	5.66 ± 0.00^Aa^	5.77 ± 0.01^Db^	6.07 ± 0.01^Dc^	6.48 ± 0.00^Dd^
T4	5.66 ± 0.00^Aa^	5.75 ± 0.00^Db^	5.97 ± 0.01^Ec^	6.42 ± 0.01^Ed^
T5	5.66 ± 0.00^Aa^	5.69 ± 0.00^Eb^	5.88 ± 0.01^Fc^	6.31 ± 0.01^Fd^

*Note:* Lowercase letters in each row and uppercase letters in each column indicate a significant difference (*p* < 0.05, Duncan′s test). Data are mean (*n* = 3) ± SD.

#### 3.3.3. TBARS (Lipid Oxidation)

TBARSs increased throughout storage, but films with EPE and BA significantly reduced lipid oxidation (Table 5). By Day 14, TBARS in T5 remained at 0.61 mg MDA/kg compared to 0.87 mg MDA/kg in the control (*p* < 0.05). This effect reflects both the oxygen barrier properties of CNC/PVA and the radical‐scavenging capacity of EPE and BA. Summary of TBARS values (mg MDA/kg) and estimated effect sizes (Cohen′s *d*) for treatment groups compared to control at Day 14 are provided in Table S4.

**Table 5:** Changes in TBARS content of fish filets during 14 days (mg MDA/kg).

**Sample**	**Day 1**	**Day 5**	**Day 9**	**Day 14**
C	0.11 ± 0.00^Aa^	0.23 ± 0.00^Ab^	0.56 ± 0.00^Ac^	0.87 ± 0.00^Ad^
T1	0.11 ± 0.00^Aa^	0.19 ± 0.00^Bc^	0.50 ± 0.00^Bc^	0.79 ± 0.00^Bd^
T2	0.12 ± 0.00^Aa^	0.16 ± 0.00^Cb^	0.45 ± 0.00^Cc^	0.71 ± 0.00^Cd^
T3	0.11 ± 0.00^Aa^	0.15 ± 0.00^Db^	0.35 ± 0.00^Dc^	0.65 ± 0.00^Dd^
T4	0.11 ± 0.00^Aa^	0.14 ± 0.00^Eb^	0.32 ± 0.00^Ec^	0.63 ± 0.00^Ed^
T5	0.11 ± 0.00^Aa^	0.13 ± 0.00^Eb^	0.30 ± 0.00^Fc^	0.61 ± 0.00^Fd^

*Note:* Lowercase letters in each row and uppercase letters in each column indicate a significant difference (*p* < 0.05, Duncan′s test). Data are mean (*n* = 3) ± SD.

#### 3.3.4. Microbiological Quality

Initial microbial loads were comparable across treatments. Counts of psychrotrophs, mesophiles, and Enterobacteriaceae increased with storage time but were consistently lower in coated groups (Table 6). By Day 14, the control exceeded the spoilage threshold of 7 log CFU/g (ICMSF), while T5 remained below this limit (psychrotrophs: 5.61 log CFU/g; mesophiles: 8.15 log CFU/g; and Enterobacteriaceae: 5.22 log CFU/g). These findings demonstrate both antimicrobial efficacy and regulatory compliance.

**Table 6:** Microbial quality of fish filets during 14 days (log CFU/g).

**Sample**	**Day 1**	**Day 5**	**Day 9**	**Day 14**
**Psychrotrophic bacteria**				
C	3.14 ± 0.01^Aa^	3.38 ± 0.00^Ab^	5.34 ± 0.00^Ac^	7.87 ± 0.01^Ad^
T1	3.15 ± 0.00^Aa^	3.35 ± 0.01^Bb^	4.85 ± 0.02^Bc^	6.79 ± 0.02^Bd^
T2	3.15 ± 0.03^Aa^	3.30 ± 0.01^Cb^	4.47 ± 0.04^Cc^	6.71 ± 0.00^Cd^
T3	3.15 ± 0.02^Aa^	3.27 ± 0.00^Db^	4.23 ± 0.01^Dc^	6.65 ± 0.00^Dd^
T4	3.15 ± 0.02^Aa^	3.21 ± 0.02^Eb^	3.91 ± 0.02^Ec^	6.63 ± 0.01^Ed^
T5	3.15 ± 0.01^Aa^	3.21 ± 0.00^Eb^	3.84 ± 0.01^Fc^	5.61 ± 0.02^Fd^
**Mesophilic bacteria**				
C	3.35 ± 0.01^Aa^	4.39 ± 0.00^Ab^	6.28 ± 0.00^Ac^	9.07 ± 0.01^Ad^
T1	3.34 ± 0.01^Aa^	4.35 ± 0.00^Bb^	6.14 ± 0.00^Bc^	8.74 ± 0.04^Bd^
T2	3.34 ± 0.01^Aa^	4.28 ± 0.01^Cb^	5.34 ± 0.00^Cc^	8.38 ± 0.00^Cd^
T3	3.34 ± 0.01^Aa^	4.22 ± 0.00^Db^	5.28 ± 0.01^Dc^	8.26 ± 0.01^Dd^
T4	3.34 ± 0.00^Aa^	4.20 ± 0.01^Eb^	5.23 ± 0.01^Ec^	8.20 ± 0.01^Ed^
T5	3.34 ± 0.00^Aa^	4.19 ± 0.01^Eb^	5.21 ± 0.01^Ec^	8.15 ± 0.01^Ed^
**Enterobacteriaceae**				
C	3.34 ± 0.00^Aa^	4.13 ± 0.01^Fb^	5.08 ± 0.02^Fc^	8.05 ± 0.01^Fd^
T1	2.53 ± 0.01^Aa^	3.13 ± 0.00^Ab^	5.21 ± 0.00^Ac^	6.35 ± 0.01^Ad^
T2	2.47 ± 0.01^Aa^	3.05 ± 0.00^Bb^	5.15 ± 0.00^Bc^	6.27 ± 0.04^Bd^
T3	2.44 ± 0.01^Aa^	2.95 ± 0.01^Cb^	4.32 ± 0.00^Cc^	5.61 ± 0.00^Cd^
T4	2.39 ± 0.01^Aa^	2.89 ± 0.00^Db^	4.24 ± 0.01^Dc^	5.37 ± 0.01^Dd^
T5	2.37 ± 0.00^Aa^	2.79 ± 0.01^Eb^	4.01 ± 0.01^Ec^	5.22 ± 0.01^Ed^

*Note:* Lowercase letters in each row and uppercase letters in each column indicate a significant difference (*p* < 0.05, Duncan′s test). Data are mean (*n* = 3) ± SD. Microbial methods followed ISO 4833‐1:2013 and ISO 21528‐2:2017.

##### 3.3.4.1. Sensory Analysis

Sensory assessment confirmed improved quality retention in coated samples. Filets wrapped in nanocomposite films, especially those with 3% EPE and BA, consistently scored higher for color, odor, texture, and overall acceptability compared to controls (*p* < 0.05). These results validate the films′ dual benefit of physicochemical protection and preservation of desirable sensory attributes during chilled storage.

## 4. Discussion

This study demonstrates the successful development and application of biodegradable CNC/PVA films incorporating EPE and BAs to enhance the shelf life of *R. frisii kutum* filets.

### 4.1. Interpretation and Contextualization of Findings

Incorporation of EPE and BA into CNC/PVA films markedly improved fish preservation, as indicated by significantly lower TVB‐N, TBARS, pH, and microbial counts over 14 days. Sensory evaluation further confirmed the films′ capacity to maintain desirable attributes such as color, odor, and texture, underscoring their practical value as active packaging materials.

These findings build upon previous studies employing plant‐derived additives in biopolymer films. For example, Arfat et al. [22] and Maghami et al. [23] reported extended shelf life in fish filets using basil‐ and fennel‐enriched nanocomposite films, respectively. While consistent with these results, our study uniquely demonstrates the synergistic effect of *E. planum* polyphenols and BAs, both of which exhibit potent antioxidant and antimicrobial properties.

The reduction in lipid oxidation can be attributed to the complementary roles of the film components. PVA primarily provides structural support with negligible antioxidant activity, while CNCs reduce oxygen permeability, indirectly slowing oxidation. EPE supplies polyphenols (e.g., rutin and chlorogenic acid) that act as radical scavengers and metal chelators, and BA contributes anthocyanins—particularly delphinidin derivatives—known for their strong radical‐scavenging capacity. Together, these components act synergistically to significantly reduce TBARS values in coated samples [17, 24].

### 4.2. Mechanisms and Synergy

The antimicrobial effects of the films likely operate via a dual mechanism. First, EPE polyphenols and BAs directly inhibit microbial growth by disrupting cell walls, compromising membrane integrity, and interfering with enzyme activity, consistent with prior reports on plant‐derived antimicrobials [25, 26]. Second, the CNC/PVA matrix enhances barrier properties by limiting oxygen and water vapor transmission, thereby restricting conditions favorable to microbial proliferation [9, 27]. This synergy was most evident in the 3% EPE formulation, where both immediate antimicrobial activity and extended preservation were observed [25, 26].

**Figure 3:**
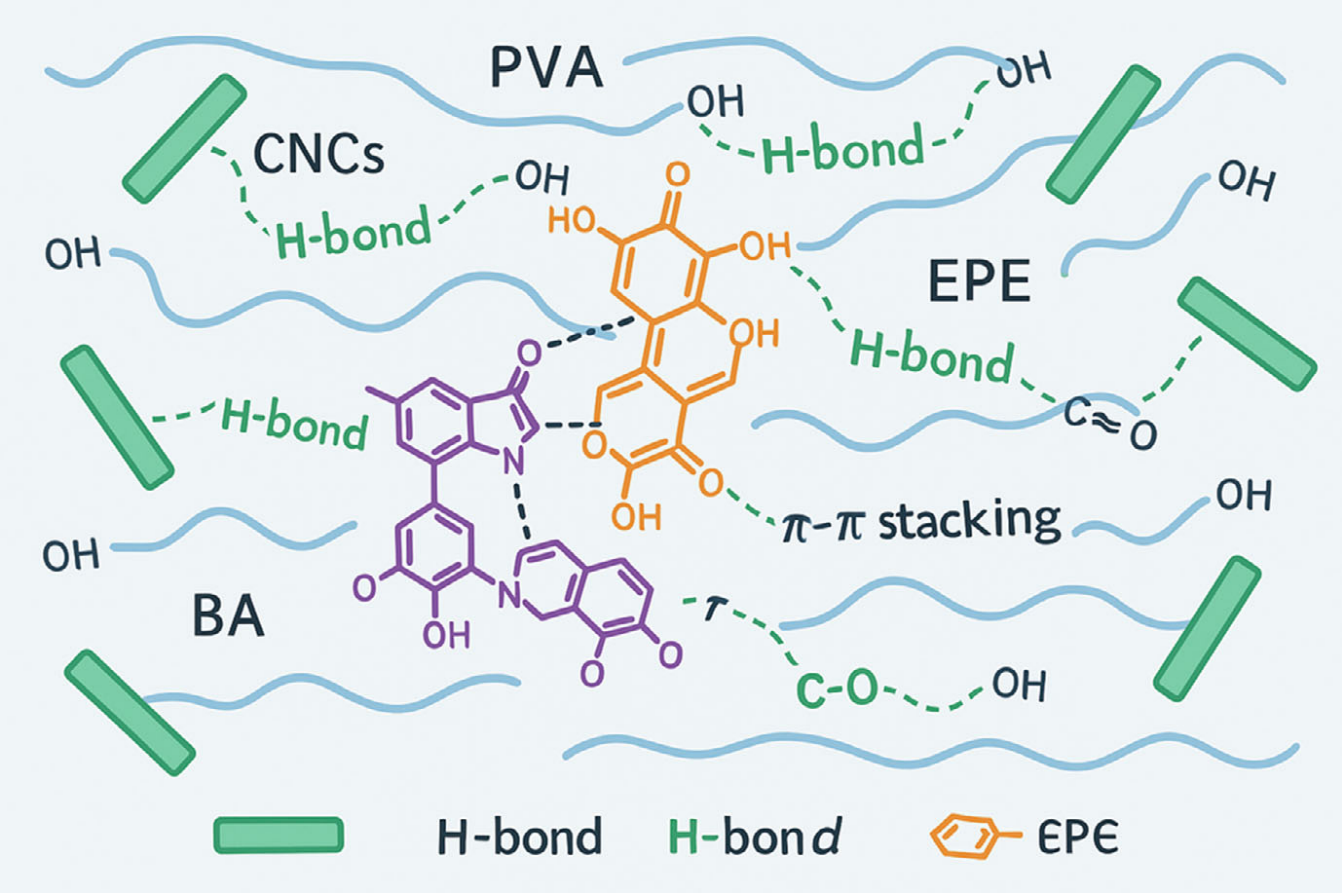
Schematic of molecular interactions within CNC/PVA/EPE/BA films. PVA chains (flexible backbones with –OH groups) form hydrogen bonds with CNCs and polyphenols. EPE compounds interact via hydrogen bonding, while BAs form both hydrogen bonds and *π*–*π* stacking interactions, collectively improving stability and functionality.

Film performance arises from multiple weak molecular interactions. Hydrogen bonding occurs between hydroxyl groups of PVA/CNC and the phenolic hydroxyls of EPE and BA [27], while *π*–*π* stacking between anthocyanins and aromatic polyphenols stabilizes bioactives within the matrix. Although covalent bonding is unlikely, these cumulative noncovalent interactions enhance miscibility, reduce phase separation, and reinforce both barrier and antimicrobial functions [9]. A schematic illustration of these interactions is provided in Figure 3.

Although EPE stability was not directly quantified, the observed retention of antioxidant and antimicrobial activity during storage suggests at least partial preservation of active compounds. Polyphenols are inherently prone to oxidative, thermal, and photolytic degradation, but incorporation into polymeric matrices such as CNC and PVA is known to improve stability by shielding them from environmental stressors [28–31]. Nevertheless, direct stability assays (e.g., HPLC profiling during storage) are warranted in future work.

### 4.3. Comparison With Existing Literature

While numerous plant‐based extracts have been explored in biopolymer films, the EPE/BA combination is unique in both composition and functional synergy. EPE is rich in rutin, chlorogenic acid, and rosmarinic acid, which differ structurally and mechanistically from compounds in more commonly studied extracts (e.g., basil, thyme, and green tea) [32]. BA provides anthocyanins with strong radical‐scavenging capacity and color stability. Together, these extracts may support both immediate and sustained release of bioactives, yielding superior preservation effects compared to single‐additive systems.

A comparative overview of recent extract‐based films (Table 7) underscores this distinction. Many previous studies reported modest improvements in oxidative stability or microbial inhibition, often limited by weak barrier properties or short‐term bioactivity [33–37]. In contrast, our CNC/PVA films containing EPE and BA achieved extended shelf life (14 days), ~30% TBARS reduction, and significant microbial inhibition, highlighting the added value of synergistic integration with CNC reinforcement.

**Table 7:** Comparative studies on extract‐enriched active films for food preservation.

**Study/material**	**Bioactive additive(s)**	**Target food**	**Key outcomes**	**Reference**
Chitosan/gelatin films	Catechin + pomegranate extract	Fish filets	Improved microbial control, extended shelf life by 3–4 days	[35]
Fish myofibrillar protein/chitosan films	Rosemary extract	Grass carp filets	Reduced lipid oxidation, delayed spoilage	[36]
Carrageenan films	Olive leaf extract	Lamb meat	Antioxidant activity, improved preservation quality	[37]
Gelatin/carrageenan/zein films	Turmeric essential oil	Chicken meat	Shelf life extension via sustained release antioxidant effect	[38]
Biopolymer films	Green tea, oregano, and rosemary extracts	Fish products	Delayed spoilage, moderate oxidative stability	[39]
CNC/PVA films (this study)	*Eryngium planum* extract (EPE) + barberry anthocyanins (BAs)	Trout filets	Shelf life extended to 14 days, ~30% TBARS reduction, strong antimicrobial protection	This work

### 4.4. Practical Implications

The demonstrated improvements in physicochemical, microbiological, and sensory quality confirm the applicability of CNC/PVA/EPE/BA films for fish packaging. Their dual antioxidant and antimicrobial functions are particularly relevant for highly perishable products, where spoilage and oxidation are key shelf‐life limitations. Extending fish freshness to 14 days under refrigeration illustrates their commercial potential. Furthermore, their biodegradable nature aligns with sustainability goals, supporting their adoption as alternatives or complements to conventional plastics.

### 4.5. Limitations

Despite promising outcomes, several limitations must be acknowledged. First, mechanical properties such as tensile strength and elongation at break were not assessed, leaving uncertainties about film robustness in real‐world applications. Second, release kinetics and migration of bioactives were not measured, limiting the mechanistic understanding of functional performance over time. Third, biodegradability was inferred from CNC/PVA literature rather than experimentally verified through composting or life cycle assessment. Finally, the chemical stability of EPE within the films was not quantified, necessitating dedicated assays in future studies.

### 4.6. Future Directions

Future research should address these limitations by (i) conducting standardized mechanical testing, (ii) characterizing migration and release kinetics of EPE and BA, (iii) experimentally validating biodegradability under soil and composting conditions, and (iv) performing life cycle assessments to quantify environmental benefits. Moreover, integrating multivariate statistical approaches such as PCA could provide deeper insights into multidimensional quality changes, while effect size metrics (e.g., partial eta‐squared) should continue to be reported to strengthen the interpretation of treatment efficacy.

## 5. Conclusion

This study demonstrated that CNC/PVA nanocomposite films enriched with EPE and BAs effectively integrate barrier reinforcement with controlled release of natural bioactives. The films significantly improved the shelf life and sensory quality of *R. frisii kutum* filets during refrigerated storage, maintaining spoilage indices below EU regulatory thresholds.

The incorporation of EPE and BA endowed the films with dual antioxidant and antimicrobial properties, confirming their value as active packaging systems. Beyond fish preservation, these films hold potential for broader application in seafood and other highly perishable foods where extending freshness without synthetic additives is critical.

While further investigations are needed—particularly on mechanical properties, biodegradability, large‐scale production, and regulatory validation—the present findings highlight the promise of CNC/PVA/EPE/BA films as sustainable and functional alternatives to conventional plastic packaging in the food industry.

## Ethics Statement

The study received ethical approval from the Shahrekord Branch, Islamic Azad University of the Ethics Committee (Approval Number: IAUSH‐2024‐8136) before the commencement of the research.

## Consent

Written informed consent was obtained from all participants prior to their inclusion in the study.

## Disclosure

No individuals or third‐party services were involved in the research or manuscript preparation who are not listed as authors or acknowledged in the manuscript.

## Conflicts of Interest

The authors declare no conflicts of interest.

## Author Contributions

BDP: data curation, methodology, and writing—original draft preparation. AS, ER, and ZM: supervision of the entire project, conceptualization, and writing—reviewing and editing. ZM and RSC: methodology and writing—reviewing and editing. RSC: assisting with statistical analyses and interpretation of the results and editing of the final manuscript.

## Funding

No funding was received for this manuscript.

## Supporting information


**Supporting Information** Additional supporting information can be found online in the Supporting Information section. Table S1: Reported mechanical properties of CNC/PVA‐based films in the literature. Table S2: Summary of TVB‐N levels and estimated effect sizes (Cohen′s *d*) for treatment groups compared to control at Day 14. Table S3: Summary of pH values and estimated effect sizes (Cohen′s *d*) for treatment groups compared to control at Day 14. Table S4: Summary of TBARS values (mg MDA/kg) and estimated effect sizes (Cohen′s *d*) for treatment groups compared to control at Day 14.

## Data Availability

The data that support the findings of this study are available from the corresponding author upon reasonable request.
