# Supplementary material for: Effect of a Biodegradable Cellulose Nanocrystal Film Containing Eryngium planum Extract and Barberry Anthocyanin on the Shelf Life of Rutilus frisii kutum Filets
Source: Int J Food Sci. 2025 Nov 21;2025:8816376. doi: 10.1155/ijfo/8816376 (PMC12635588; doi:10.1155/ijfo/8816376)
Supplement: Supplementary file 1 — Supporting Information Additional supporting information can be found online in the Supporting Information section. Table S1: Reported mechanical properties of CNC/PVA‐based films in the literature. Table S2: Summary of TVB‐N levels and estimated effect sizes (Cohen′s d) for treatment groups compared to control at Day 14. Table S3: Summary of pH values and estimated effect sizes (Cohen′s d) for treatment groups compared to control at Day 14. Table S4: Summary of TBARS values (mg MDA/kg) and estimated effect sizes (Cohen′s d) for treatment groups compared to control at Day 14. [file IJFO-2025-8816376-s001.docx]

**Table S1.** Reported Mechanical Properties of CNC/PVA-Based Films in Literature

| **Reference** | **CNC content (% w/w)** | **PVA content** | **Tensile Strength (MPa)** | **Elongation at Break (%)** | **Young’s Modulus (GPa)** | **Notes** |
| --- | --- | --- | --- | --- | --- | --- |
| Arfat et al., 2015 [24] | 2–5% CNC | PVA matrix | 32–45 | 110–140 | 1.1–1.6 | Improved strength vs. neat PVA |
| Maghami et al., 2019 [25] | 3% CNC | PVA matrix | 38.2 ± 1.2 | 102 ± 4 | 1.4 ± 0.2 | Good flexibility; bioactive-loaded |
| Zhang et al., 2023 [8] | 4% CNC | PVA | 35.4 ± 2.1 | 108 ± 3 | 1.5 ± 0.1 | Reported improved antioxidant stability |

Mechanical properties (tensile strength, elongation at break) were not evaluated in this study. However, reported values from similar CNC/PVA films in the literature (Supplementary Table S1) indicate that such systems generally achieve tensile strengths of 28–47 MPa with elongation at break of 95–140%, suggesting suitability for packaging applications. Future work should experimentally confirm film robustness.

**Table S2.** Summary of TVB-N levels and estimated effect sizes (Cohen’s d) for treatment groups compared to control at day 14

| **Group** | **Mean TVB-N (mg N/100g)** | **SD** | **n** | **Effect Size vs Control (Cohen’s d)** |
| --- | --- | --- | --- | --- |
| Control | 53.10 | 0.47 | 3 | - |
| T1 | 47.88 | 0.22 | 3 | 14.23 |
| T2 | 47.04 | 0.22 | 3 | 16.51 |
| T3 | 45.45 | 0.13 | 3 | 22.19 |
| T4 | 44.24 | 0.22 | 3 | 24.15 |
| T5 | 43.68 | 0.22 | 3 | 25.38 |

The results indicate that treatments T3–T5 exerted large effect sizes relative to control, supporting their strong preservative potential.

**Table S3.** Summary of pH values and estimated effect sizes (Cohen’s d) for treatment groups compared to control at day 14.

| **Group** | **Mean pH (Day 14)** | **SD** | **n** | **Effect Size vs Control (Cohen’s d)** |
| --- | --- | --- | --- | --- |
| Control | 6.87 | 0.01 | 3 | - |
| T1 | 6.61 | 0.00 | 3 | 16.44 |
| T2 | 6.55 | 0.01 | 3 | 16.00 |
| T3 | 6.48 | 0.00 | 3 | 24.67 |
| T4 | 6.42 | 0.01 | 3 | 22.50 |
| T5 | 6.31 | 0.01 | 3 | 28.00 |

Progressive reduction in pH values in EPE/BA films, particularly in T4 and T5, showed very large effect sizes, confirming significant suppression of spoilage-related alkalinity.

**Table S4.** Summary of TBARS values (mg MDA/kg) and estimated effect sizes (Cohen’s d) for treatment groups compared to control at day 14.

| **Group** | **Mean TBARS (mg MDA/kg, Day 14)** | **SD** | **n** | **Effect Size vs Control (Cohen’s d)** |
| --- | --- | --- | --- | --- |
| Control | 0.87 | 0.00 | 3 | - |
| T1 | 0.79 | 0.00 | 3 | 8.00 |
| T2 | 0.71 | 0.00 | 3 | 16.00 |
| T3 | 0.65 | 0.00 | 3 | 22.00 |
| T4 | 0.63 | 0.00 | 3 | 24.00 |
| T5 | 0.61 | 0.00 | 3 | 26.00 |

Marked decreases in lipid oxidation were observed, with T3–T5 treatments yielding extremely large effect sizes, highlighting the antioxidant efficacy of EPE/BA-incorporated films.
